# Extreme In Situ Liver Surgery Under Total Vascular Exclusion with Right Hepatic Vein and Inferior Vena Cava Grafts for an Intrahepatic Cholangiocarcinoma

**DOI:** 10.1245/s10434-022-12787-4

**Published:** 2022-12-03

**Authors:** Victor Lopez-Lopez, Paula Gomez Valles, Carlos García Palenciano, Sergio Canovas, Asunción López Conesa, Roberto Brusadin, Ricardo Robles-Campos

**Affiliations:** 1 Department of Surgery and Liver and Pancreas Transplantation, IMIB-Arrixaca, Virgen de la Arrixaca Clinic and University, Murcia, Spain; 2 Department of Anesthesiology, IMIB-Arrixaca, Virgen de la Arrixaca Clinic and University, Murcia, Spain; 3 Department of Cardiac Surgery, IMIB-Arrixaca, Virgen de la Arrixaca Clinic and University, Murcia, Spain

## Abstract

In this multimedia article, we demonstrate an extreme in situ liver surgery under total vascular exclusion with right hepatic vein and inferior vena cava grafts for an intrahepatic cholangiocarcinoma in a centre with experience in highly complex hepatobiliary surgery and liver transplantation. This surgical approach after neoadjuvant chemotherapy provides an opportunity for surgical salvage in patients with large tumors invading the hepatocaval confluence. This patient was considered unresectable at another hospital and referred to our unit. We performed an accurate preoperative assessment with new generation 3D modelling to plan the type of vascular reconstruction that would allow adequate hepatic venous outflow and the volume of the future liver remnant sufficient to avoid postoperative liver failure. For hemodynamic management of the patient, we performed a total hepatic vascular exclusion with veno-venous bypass without intraoperative adverse events. We used a cryopreserved carotid artery graft after previously planning the most appropriate diameter and length for right hepatic vein reconstruction. The inferior vena cava was reconstructed with gore-tex graft. During the hospital stay there were no postoperative complications. The patient is free of disease. We conclude that patients with advanced malignant liver disease should always be referred to highly specialized liver surgery centers to assess the most appropriate oncological management and the possibility of surgical resectability.

**Supplementary Information:**

The online version contains supplementary material available at 10.1245/s10434-022-12787-4.

There are different scenarios in liver surgery in which the location of the tumor makes it difficult to achieve a tumor-free resection. One of these surgical and hemodynamic challenges is the invasion of the three hepatic veins and inferior vena cava by a malignant primary tumor. Experience in liver transplantation and complex hepatobiliary surgery together with advances in anesthetic management and preoperative planning have allowed initially unresectable tumors to be safely removed.

## Surgical Approach

We present the case of a 52-year-old man who was diagnosed with intrahepatic cholangiocarcinoma with vascular invasion. The patient was referred from another hospital. The computed tomography (CT) and magnetic resonance imaging (MRI) reported a mass of approximately 7.89 cm in the left hepatic lobe with peripheral expansion toward segments 5-8. The tumor had the following relationships with venous structures: (1) invasion of the left and middle hepatic vein (at its confluence with the cava); (2) contact with the confluence of the anterior branch of the right hepatic vein; (3) intrahepatic vena cava infiltration over a length of approximately 7.64 cm; (4) a free inferior hepatic vein; and (5) portal vein with minimal contact at the level of the extrahepatic portal vein. The CT and an 18-FDG PET-CT ruled out the presence of distant disease and lymph node metastases. A next-generation three-dimensional modelling software was used for surgical planning, volumetry, and venous drainage calculations.

The technique planned was an extended left hepatectomy with reconstruction of the vena cava with a Gore-Tex prosthesis above the inferior hepatic vein that was not infiltrated and a repair of the right hepatic vein with a carotid graft obtained previously from a tissue bank. Surgery was planned under total vascular exclusion with a veno-venous bypass. During occlusion, in situ hypothermic perfusion was performed through the left portal vein. Operative time was 420 minutes with blood loss of 400 ml and without transfusions. The patient was discharged on the tenth postoperative day without complications, and he is 1-year disease-free.

## Discussion

The first decision to be made in these cases is whether to perform the liver resection ex vivo or in vivo.^1,2^ Our group decided to perform it in vivo to avoid the additional risk of warm ischemia and possible complications associated with portal, arterial, and biliary anastomoses. Second, although our group has experience in liver resections within situ hepatic vein reconstruction with partial vascular occlusion, in this case we decided to opt for total vascular occlusion with placement of a veno-venous bypass because the patient needed both right hepatic vein and vena cava reconstruction.^3,4^ For the right hepatic vein graft, a graft from the tissue bank was chosen to avoid the complications that can occur with an autologous graft.^5^

Highly specialized units in hepatobiliary surgery and transplantation with experience in extreme liver surgery are crucial to be able to operate on patients who are considered unresectable in other centers. Today, these patients must be evaluated by expert committees in complex cases to offer the possibility of a safe surgery with good oncologic outcomes.

## Supplementary Information

Below is the link to the electronic supplementary material.

Supplementary file1 (MP4 194250 kb)

## Data Availability

Not applicable.
